# New-Onset Kidney Diseases after COVID-19 Vaccination: A Case Series

**DOI:** 10.3390/vaccines10020302

**Published:** 2022-02-16

**Authors:** Jeong-Hoon Lim, Mee-Seon Kim, Yong-Jin Kim, Man-Hoon Han, Hee-Yeon Jung, Ji-Young Choi, Jang-Hee Cho, Chan-Duck Kim, Yong-Lim Kim, Sun-Hee Park

**Affiliations:** 1 Division of Nephrology, Department of Internal Medicine, School of Medicine, Kyungpook National University, Kyungpook National University Hospital, Daegu 41944, Korea; jh-lim@knu.ac.kr (J.-H.L.); hy-jung@knu.ac.kr (H.-Y.J.); jy-choi@knu.ac.kr (J.-Y.C.); jh-cho@knu.ac.kr (J.-H.C.); drcdkim@knu.ac.kr (C.-D.K.); ylkim@knu.ac.kr (Y.-L.K.); 2 Department of Pathology, School of Medicine, Kyungpook National University, Kyungpook National University Hospital, Daegu 41944, Korea; kimm2342@gmail.com (M.-S.K.); yyjjkim@knu.ac.kr (Y.-J.K.); mhhan1@knu.ac.kr (M.-H.H.)

**Keywords:** COVID-19, vaccination, kidney disease, kidney biopsy, IgA nephropathy, minimal change disease, thrombotic microangiopathy, tubulointerstitial nephritis

## Abstract

Various vaccines against COVID-19 have been developed and proven to be effective, but their side effects, especially on kidney function, are not yet known in detail. In this study, we report the clinical courses and histopathologic findings of new-onset kidney diseases after COVID-19 vaccination as confirmed via kidney biopsy. Five patients aged 42 to 77 years were included in this study, and baseline kidney function was normal in all patients. The biopsy-proven diagnosis indicated newly developed kidney diseases: (1) IgA nephropathy presenting with painless gross hematuria, (2) minimal change disease presenting with nephrotic syndrome, (3) thrombotic microangiopathy, and (4) two cases of acute tubulointerstitial nephritis presenting with acute kidney injury. Individualized treatment was applied as per disease severity and underlying pathology, and the treatment outcomes of all patients were improved. Since this is not a controlled study, the specific pathophysiologic link and causality between the incidence of kidney diseases and COVID-19 vaccination are difficult to confirm. However, clinicians need to consider the possibility that kidney diseases may be provoked by vaccines in patients who have renal symptoms.

## 1. Introduction

The coronavirus disease 2019 (COVID-19) pandemic, which is caused by severe acute respiratory syndrome coronavirus 2 (SARS-CoV-2) infection, is still ongoing globally. Although therapeutic agents that successfully suppress COVID-19 have not yet been developed, several vaccines against SARS-CoV-2 can help in the prevention of COVID-19 and alleviation of the disease’s severity.

Two main types of vaccine are currently used to prevent COVID-19: (1) mRNA vaccines, such as BNT162b2 (Pfizer-BioNTech) and mRNA-1273 (Moderna); and (2) vectored vaccines, such as AZD1222 (Oxford-AstraZeneca) and Ad26.COV2.S (Janssen). Several clinical studies have demonstrated that these vaccines have excellent protective effects against COVID-19, but their side effects are not yet well-known [1,2]. To prove a causal relationship between side effects after vaccination is difficult, so the side effects of vaccines are occasionally overlooked.

Several studies have reported on kidney diseases that occurred after COVID-19 vaccinations, but the number of cases was few, and the histopathologic abnormalities and clinical courses were heterogeneous [3,4]. This limited information could delay the diagnosis and treatment of renal side effects after COVID-19 vaccination, resulting in a poor prognosis. Therefore, in this study, we present five cases of new-onset renal pathology confirmed by kidney biopsy from a tertiary hospital in Korea, which were clinically presented as acute kidney injury or urinary abnormalities after COVID-19 vaccination. Table 1 shows the details of the cases.

## 2. Case Presentation

### 2.1. IgA Nephropathy Presenting with Painless Gross Hematuria

**Figure 1:**
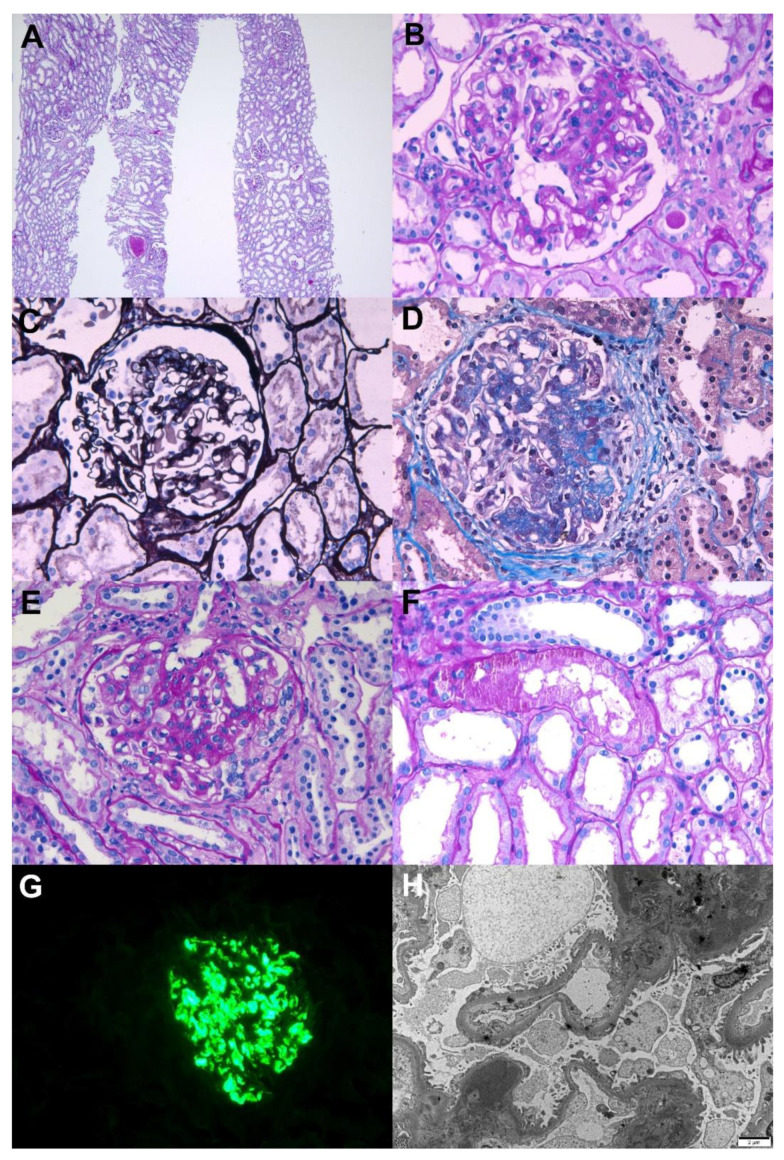
Pathological findings of IgA nephropathy. Light microscopy: (**A**) In low magnification, no tubular atrophy or interstitial fibrosis was present (periodic acid–Schiff, ×40). (**B**) The glomerulus shows mesangial hypercellularity with increased mesangial matrix (periodic acid–Schiff, ×400). (**C**) The glomerulus shows endocapillary hypercellularity with focal loss of capillary penetrations (periodic acid methenamine silver, ×400). (**D**) The glomerulus shows segmental glomerulosclerosis (Masson’s trichrome, ×400). (**E**) The glomerulus shows cellular crescent with endocapillary hypercellularity (periodic acid–Schiff, ×400). (**F**) The picture shows protein reabsorption vacuoles in tubules, which is evidence of proteinuria (periodic acid–Schiff, ×400). Immunofluorescence: (**G**) Strong IgA mesangial positivity in the immunofluorescence stain (×400). Electron microscopy: (**H**) Numerous large electron-dense deposits in the mesangial areas with focal foot process effacement in the capillary surface were observed (×6000, 80 kv).

A healthy 42-year-old woman presented with first-onset painless gross hematuria and proteinuria. She had no family history of kidney diseases. She had received two doses of the mRNA-1273 vaccine with a 4-week interval. One day after receiving the second dose, she developed dark reddish urine. Gross hematuria disappeared within several days, but follow-up urinalysis showed persistent microscopic hematuria and proteinuria. Based on the laboratory data, the serum creatinine level was 0.47 mg/dL, and the 24 h urinary protein level was 1.7 g/day. Serologic evaluation revealed no specific abnormalities in viral markers or autoantibodies. A kidney biopsy was performed 8 weeks after the vaccination, and the results are shown in Figure 1. Light microscopy revealed mesangial hypercellularity with increased mesangial matrix, cellular crescent, segmental sclerosis, and endocapillary proliferation. Immunofluorescence revealed 3+ diffuse mesangial staining for IgA. Electron microscopy was also consistent with IgA nephropathy, and the Oxford MEST-C classification was M0E1C1S1T0. She was treated with losartan (50 mg), and the spot urine protein-to-creatinine ratio was decreased to 0.7 g/g.

### 2.2. Minimal Change Disease Presenting with Nephrotic Syndrome

**Figure 2:**
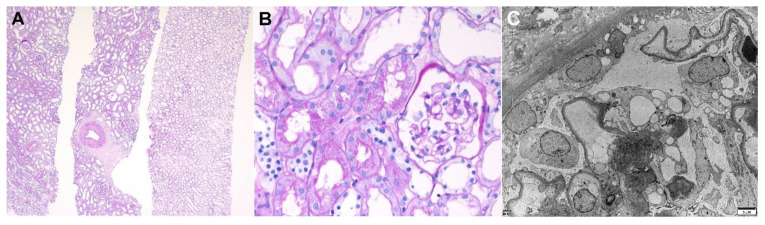
Pathologic findings of minimal change disease. Light microscopy: (**A**) In low magnification, no tubular atrophy or interstitial fibrosis was present (hematoxylin and eosin, ×40). (**B**) The picture shows a normally appearing glomerulus with protein reabsorption granules in tubules (hematoxylin and eosin, ×400). Electron microscopy: (**C**) Diffuse podocyte foot process effacement with microvillous transformation without electron-dense deposits were observed. The thickness of glomerular basememt membrane was normal (×2500, 80 kv).

A healthy 51-year-old man with no significant past medical history was referred to our clinic because of severe generalized edema. The edema had occurred 7 days after receiving the first dose of Ad26.COV.2 vaccine, and he gained 14 kg for 3 weeks after vaccination. The laboratory examinations revealed a serum creatinine level of 1.54 mg/dL, an albumin level of 1.6 g/dL, and a 24 h urinary protein level of 8.6 g/day. The serologic markers for glomerulonephritis, including viral markers, were all negative. A kidney biopsy was performed 33 days after the vaccination, and the results are shown in Figure 2. Diffuse effacement of the podocyte foot processes was observed on the electron microscopy, and no specific findings from the light microscopy and immunofluorescence were recorded. He was treated with a high-dose steroid treatment (1 mg/kg prednisone), and complete remission was achieved after 3 weeks of treatment.

### 2.3. Thrombotic Microangiopathy Presenting with Acute Kidney Injury

**Figure 3:**
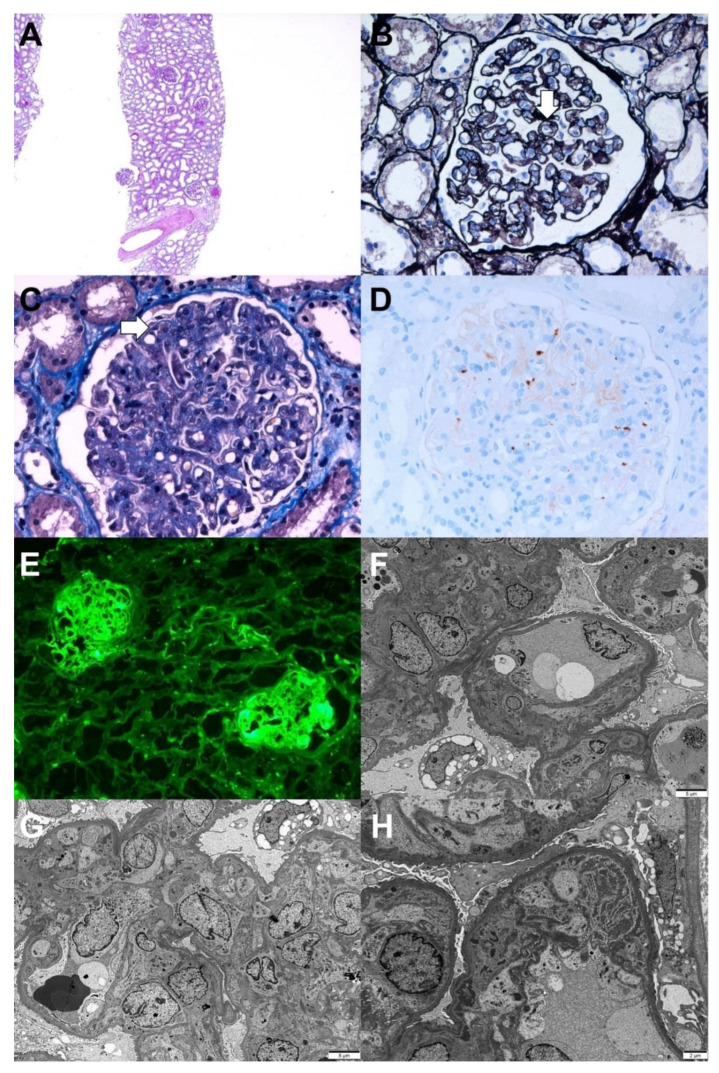
Pathologic findings of chronic thrombotic microangiopathy. Light microscopy: (**A**) In low magnification, no tubular atrophy or interstitial fibrosis was present (hematoxylin and eosin, ×40). (**B**) The picture shows the duplication of the capillary basement membrane (arrow) (periodic acid methenamine silver, ×400). (**C**) The glomerular lumina were occluded due to the thickening of the glomerular capillary wall with a few fibrin thrombi (arrow) (Masson’s trichrome, ×400). (**D**) Immunohistochemical stain for CD61 (platelet glycoprotein GPIIIa) shows positivity against platelet thrombi (CD61, ×400). Immunofluorescence: (**E**) Strong positive staining for fibrinogen in the glomerulus was observed(×100). Electron microscopy: (**F**) Glomerular basement membrane duplication with cellular interpositions was observed (×3000, 80 kv). (**G**) Glomerular endothelial swelling and hypertrophy with occlusion of the lumens (glomerular endotheliosis) and diffuse foot process effacements along the capillary surfaces were observed (×3000, 80 kv). (**H**) Deposition of fibrinogen in the glomerular intracapillary area with entrapped cellular debris was noticed (×5000, 80 kv).

A 69-year-old woman with type 2 diabetes mellitus visited the emergency room because of azotemia and thrombocytopenia. She received her first dose of the AZD1222 vaccine 5 days prior to her visit, and general weakness and gastrointestinal discomfort were observed 2 days later. Based on the laboratory examination, the hemoglobin level was 8.5 g/dL, platelet count 38 × 10^9^/L, serum creatinine level 3.69 mg/dL, and spot urine protein-to-creatinine ratio 2.7 g/g. The serologic evaluation for glomerulonephritis was all-negative. A kidney biopsy was performed 2 weeks after the vaccination, and the results are shown in Figure 3. Light microscopy revealed diffuse thickening of the capillary wall with capillary loop doubling and hyaline thrombi in the glomeruli with intact tubule and interstitium. Immunofluorescence for fibrinogen revealed a strong positivity in the glomeruli. Electron microscopy revealed duplications of the glomerular basement membranes (GBM), endothelial swelling and hypertrophy with occlusion of the lumens, and glomerular intracapillary fibrin deposition with entrapped cellular debris. The pathological findings indicated chronic thrombotic microangiopathy (TMA). In addition, the ADAMTS13 activity was 68.9%, and the Shiga toxin result was negative. Acute kidney injury and thrombocytopenia gradually and spontaneously resolved, the serum creatinine level decreased to 0.65 mg/dL, and the spot urine protein-to-creatinine ratio was 1.0 g/g 8 weeks after the vaccination.

### 2.4. Acute Tubulointerstitial Nephritis Presenting with Acute Kidney Injury

**Figure 4:**
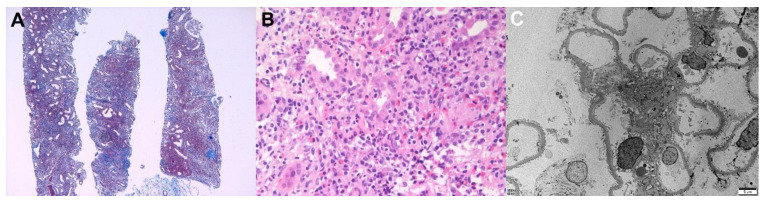
Pathologic findings of acute tubulointerstitial nephritis (patient 1). Light microscopy: (**A**) Massive interstitial inflammatory infiltrates with fibrosis (Masson’s trichrome, ×40) and (**B**) Mixed inflammatory cells infiltrated the tubular epithelium (tubulitis) and interstitium were observed. Large numbers of mononuclear cells, neutrophils, eosinophils, and plasma cells were present (hematoxylin and eosin, ×400). Electron microscopy: (**C**) The glomerulus showed a relatively normal appearance. No electron-dense deposits with a normal thickness of basement membrane and intact foot processes were present (×2500, 80 kv).

A 44-year-old man with a chronic hepatitis B infection and type 2 diabetes mellitus visited the nephrology clinic because of decreased renal function. His baseline serum creatinine level was 0.91 mg/dL; however, it increased to 4.94 mg/dL upon admission. He received the first dose of the mRNA-1273 vaccine 3 weeks prior to his visit, and gastrointestinal discomfort and anorexia occurred 1 day after vaccination. His general condition did not improve, so he visited a local clinic and exhibited a serum creatinine level of 4.13 mg/dL 1 week after vaccination. He did not take any nephrotoxic agents and drank enough water, but his azotemia worsened. The laboratory examinations revealed a spot urine protein-to-creatinine ratio of 1.0 g/g and no microscopic hematuria, and the serologic markers for glomerulonephritis were all negative except for hepatitis B. A kidney biopsy was performed 28 days after the vaccination, and the results are shown in Figure 4. Light microscopy revealed massive inflammatory cell infiltration in the interstitium and tubular epithelium. The inflammatory cells consisted of mononuclear cells, neutrophils, eosinophils, and plasma cells. Immunofluorescence and electron microscopy revealed normal pathology. A high-dose steroid treatment (prednisolon 60 mg/day) was applied to treat acute tubulointerstitial nephritis. Six weeks later, his serum creatinine level was decreased to 1.89 mg/dL, and his spot urine protein-to-creatinine ratio was 0.3 g/g.

**Figure 5:**
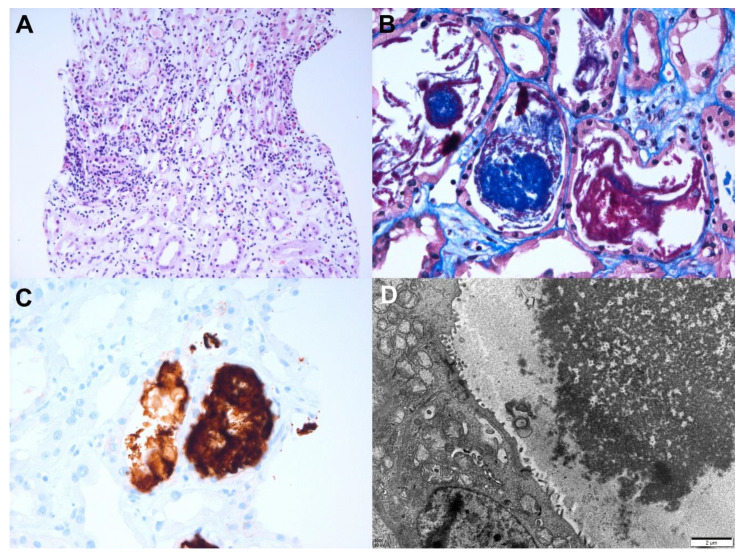
Pathologic findings of acute tubulointerstitial nephritis (patient 2). Light microscopy: (**A**) Mixed inflammatory cells infiltrated the tubular epithelium (tubulitis) and interstitium. Large numbers of mononuclear cells, neutrophils, eosinophils, and plasma cells were present (hematoxylin and eosin, ×400). (**B**) Acute tubular injury with red granular casts and string-like appearing casts was observed(Masson’s trichrome, ×400). (**C**) The casts show strong myoglobin positivity in the immunohistochemical stain (myoglobin, ×400). Electron microscopy: (**D**) The tubular casts have electron dense granules (×8000, 80 kv).

A 77-year-old woman with a chronic hepatitis B infection, hepatocellular carcinoma, and type 2 diabetes mellitus visited the emergency room, presenting with anorexia and nausea following vaccination. She received the second dose of the BNT162b2 vaccine 7 days prior to her visit. The day after vaccination, she had experienced severe nausea and vomiting, so she received intravenous fluid therapy. Her symptoms did not improve, and 1 week after the vaccination, her serum creatinine level was increased from 0.98 mg/dL to 10.75 mg/dL, and her bicarbonate level was decreased to 8.2 mmol/L. Based on the serologic examination, her muscle enzyme levels were slightly increased (creatine phosphokinase 381 U/L, lactate dehydrogenase 427 U/L, and myoglobin 1180 ng/mL), and the serologic markers were all negative except for hepatitis B surface antigen. Hemodialysis was initiated, and a kidney biopsy was performed 2 weeks after vaccination (Figure 5). Light microscopy revealed normal glomeruli, mild infiltration of lymphocytes in the interstitium, and myoglobin casts in the tubules. Electron dense casts in the tubules were also observed during the electron microscopy. No specific findings in immunofluorescence were recorded. Considering her condition, she underwent a low-dose steroid treatment (prednisolone 20 mg/day), and her kidney function gradually recovered indicating a serum creatinine level of 2.12 mg/dL 4 months after the vaccination.

## 3. Discussion

In this report, we reviewed the clinical course, treatment, and histopathologic findings of various new-onset kidney diseases following COVID-19 vaccination. This is not a controlled study, so causality between vaccinations and these diseases cannot be concluded. However, COVID-19 vaccines are known to cause new-onset or relapsing glomerular diseases due to potent immune dysregulation, and various therapeutic responses have been reported [4,5]. Therefore, special attention is required for the onset of kidney disease symptoms in recently vaccinated patients, such as foamy urine, hematuria, and edema.

IgA nephropathy is the most common glomerulonephritis identified in kidney biopsy, and it is an immune complex disease caused by mesangial IgA1 deposition with or without concurrent IgG and C3 deposits [6,7]. Although the factors causing the occurrence of IgA nephropathy have not been clearly identified, IgA nephropathy is proposed as a multi-hit disease. If patients who have a genetic predisposition (genetic variation encoding galactosylation) are exposed to subsequent triggering events, such as infection, dietary and environmental stress lead to the production of anti-glycan IgA/IgG, and IgA nephropathy will occur [8]. Klomjit et al. reported in their study that COVID-19 vaccination was associated with glomerulonephritis, and 5 out of 13 patients were diagnosed with IgA nephropathy (4 new-onset and 1 relapse) [4]. Among them, the partial nephrectomy sample of one patient before vaccination indicated IgA deposition. Therefore, IgA nephropathy after COVID-19 vaccination may be the result of a flare. Moreover, the most common symptom of IgA nephropathy after a COVID-19 vaccination was gross hematuria, and most patients showed a self-limited clinical course without immunosuppression [4]. In addition, eight cases of relapse IgA nephropathy were recorded, and all of them spontaneously recovered within 2 weeks [4].

Minimal change disease (MCD) is a glomerular disease typically characterized by nephrotic-range proteinuria, diffuse foot process effacement in electron microscopy, and no specific abnormalities in light microscopy and immunofluorescence. It has also been confirmed that podocyte injury is caused by various circulating cytokines, such as interleukin (IL)-4, 5, 9, 10, and 13, which are released by activated T lymphocytes [3,9]. Recently, a Netherlands registry study showed podocyte-associated punctate polyclonal IgG deposits in MCD after COVID-19 vaccination, so B cell activation may also contribute to the onset of MCD to some degree [10]. Typical clinical and pathologic MCD features were confirmed in our case, which may be due to the potent immune response of the COVID-19 vaccine. Timmermans et al. also reported that biopsy-proven primary podocytopathies were significantly increased after COVID-19 vaccination compared with those before the COVID-19 pandemic, and incidences of MCD have since mainly increased [10]. These findings suggest a potential link between COVID-19 vaccinations and MCD. The patients responded well to steroid treatment, as did the general MCD cases. Some new-onset or relapse MCD cases after COVID-19 vaccination were reported, and most of them responded well to the high-dose steroid treatment and showed rapid remission [4,10,11,12,13]. However, one case showed no response to the high-dose steroid treatment, and complete remission was achieved after the administration of rituximab [4].

TMA refers to a spectrum of similar disease entities featured by microangiopathic hemolytic anemia, thrombocytopenia, and acute kidney injury, and can be caused by various etiologies [14]. TMA is characterized by the wall-thickening of microvessels (arterioles and capillaries), endothelial cells swelling and separating from the GBM, and blood clots containing platelets in the lumen of microvessels [15]. These narrow the vascular lumen, especially for microvessels, and lead to occlusion. Histopathological findings vary depending on the severity and duration of the TMA. Initially, the gap between the glomerular capillary endothelial cells and the GBM widens, leading to a thickening of the capillary wall. In the chronic phase, a new GBM is formed and makes two layers of GBM. Thrombi composed of a mass of destroyed red blood cells, fibrin, and platelets in the glomerular capillaries and mesangium can be observed [15,16]. However, the pathophysiologic mechanism of TMA after COVID-19 vaccination has not yet been clarified. Fabritiis et al. also reported a case of TMA after the BNT162b2 vaccination [17]. In this case, foamy urine was observed 5 weeks after the second dose of vaccination, and complete remission was achieved after steroid pulse therapy and high-dose steroid therapy. As in the case of TMA after influenza vaccination, complement activation by immune dysregulation is presumed to be involved in its occurrence [14], and as symptoms appeared early after vaccination, T lymphocytes may be an important mediator of vaccine-related TMA.

Acute tubulointerstitial nephritis (ATIN) can be caused by a hypersensitivity reaction from the use of various vaccines or drugs [18,19,20]. The development of ATIN is suggested to be caused by a T lymphocyte-mediated injury with an aberrant innate and consequent acquired immune response [21,22,23]. Ingredients found in COVID-19 vaccines, such as mRNA and polyethylene glycol, are known to be immunogenic agents and can induce a hypersensitivity-like reaction [22,24]. Some ATIN cases after COVID-19 vaccination have been observed and responded well to steroid treatment [4,18]. The occurrence of muscle syndromes, such as myalgia, myositis, and rhabdomyolysis, is a well-known side effect of vaccination [25]. Myalgia is a common side effect of COVID-19 vaccinations, and cases of rhabdomyolysis after vaccination have also been reported [25]. The myoglobin cast itself is known to rarely cause kidney injury, but it is known to cause renal toxicity, volume depletion, ischemia, hypotension, and acidic urine, so it is important for high-risk patients to have enough water intake [26].

## 4. Conclusions

This case series provides information on new-onset renal histopathology after COVID-19 vaccination. The need for COVID-19 vaccination, including booster shots, is being emphasized globally. Although we could not confirm causality between vaccinations and these phenomena, in this time of mass vaccination, clinicians need to consider the possibility that vaccines may have provoked kidney diseases in patients who have renal symptoms.

## Figures and Tables

**Table 1:** Summary of presented cases of new-onset renal histopathology.

Pathologic Diagnosis	Age/ Sex	Clinical Presentation	Underlying Diseases	Types of Vaccine/ Manufacturer/ Dose	Symptom Onset Time/Biopsy Time after Vaccination, Days	Kidney Histopathology	Scr at Baseline, mg/dL	Scr at Biopsy, mg/dL	UPCR at Biopsy, g/gCr	Treatment	Response (Follow-Up Duration after Diagonsis)
IgA nephropathy, Haas III (M0E1C1S1T0)	42/F	Gross hematuria	None	mRNA/ Moderna (Cambridge, MA, USA)/ 2nd	1/54	LM: mesangial hypercellular with increased mesangial matrix, cellular crescent, segmental sclerosis, endocapillary proliferation IF: IgA 3+, C3 1+, kappa 2+, lambda 2+ EM: many large mesangial electron dense deposits, focal foot process loss	0.47 at 5 weeks before biopsy	0.45	1.67	RASi	PR (11 weeks)
Minimal change disease	52/M	Nephrotic syndrome	None	Vector/ Janssen (Raritan, NJ, USA)/ 1st	7/33	LM: normal glomeruli, intact tubules and interstitium IF: all negative EM: diffuse foot process loss	NA (normal)	1.96	7.12	High-dose steroid treatment	CR (31 weeks)
Chronic thrombotic microangiopathy	69/F	Acute kidney injury	Type 2 diabetes mellitus	Vector/ AstraZeneca (Cambridge, UK)/ 1st	2/14	LM: diffuse thickening of the capillary wall with capillary loop doubling, hyaline thrombi in glomeruli, intact tubules and interstitium, mild infiltration of lymphocytes in the interstitium, arterial fibrointimal thickening IF: fibrinogen 3+ in the glomeruli EM: duplications of the glomerular basement membranes with cellular interpositions, endothelial swelling and hypertrophy with occlusion of the lumens, glomerular intracapillary fibrin deposition with entrapped cellular debris, diffuse foot process loss	0.80 at 1 year before biopsy	3.69	5.20	None	SR (21 weeks)
Acute tubulointerstitial nephritis	44/M	Acute kidney injury	Type 2 diabetes mellitus, chronic hepatitis B, hyperlipidemia	mRNA/ Moderna (Cambridge, MA, USA)/ 1st	1/28	LM: normal glomeruli, mild IF/TA, massive mixed inflammatory cell infiltrates in the tubular epithelium (tubulitis) and interstitium IF: all negative EM: normal glomeruli	0.91 at 10 weeks before biopsy	4.94	1.01	High-dose steroid treatment	PR (11 weeks)
Acute tubulointerstitial nephritis with myoglobin tubular casts	77/F	Acute kidney injury	Chronic hepatitis B, hepatocellular carcinoma, type 2 diabetes mellitus	mRNA/ Pfizer (New York, NY, USA)/ 2nd	1/14	LM: normal glomeruli, mild IF/TA, infiltration of the inflammatory cells in the interstitium, myoglobin casts in the tubules IF: all negative EM: electron dense granular casts in tubules, focal foot process loss	0.98 at 12 weeks before biopsy	11.15	4.63	Low-dose steroid treatment, hemodialysis	PR (23 weeks)

Abbreviations: Scr, serum creatinine; UPCR, urine protein-to-creatinine ratio; M, mesangial hypercellularity; E, endocapillary hypercellularity; C, crescents; S, segmental glomerulosclerosis; T, tubular atrophy/interstitial fibrosis; LM, light microscopy; IF, immunofluorescence; EM, electron microcopy; RASi, renin angiotensin system inhibitor; PR, partial remission; CR, complete remission; SR, spontaneous remission; IF/TA, tubular atrophy and interstitial fibrosis.

## Data Availability

The data of this study is available from the corresponding author upon reasonable request.
